# Supplementary material for: Enhancements of electric field and afterglow of non-equilibrium plasma by Pb(ZrxTi1−x)O3 ferroelectric electrode
Source: Nat Commun. 2024 Apr 10;15:3092. doi: 10.1038/s41467-024-47230-7 (PMC11006859; doi:10.1038/s41467-024-47230-7)
Supplement: Supplementary file 1 — Supplementary Information [file 41467_2024_47230_MOESM1_ESM.pdf]

Enhancements of Electric Field and Afterglow of Non-equilibrium Plasma by $Pb(Zr_xTi_{(1-x)})O_3$ Ferroelectric Electrode

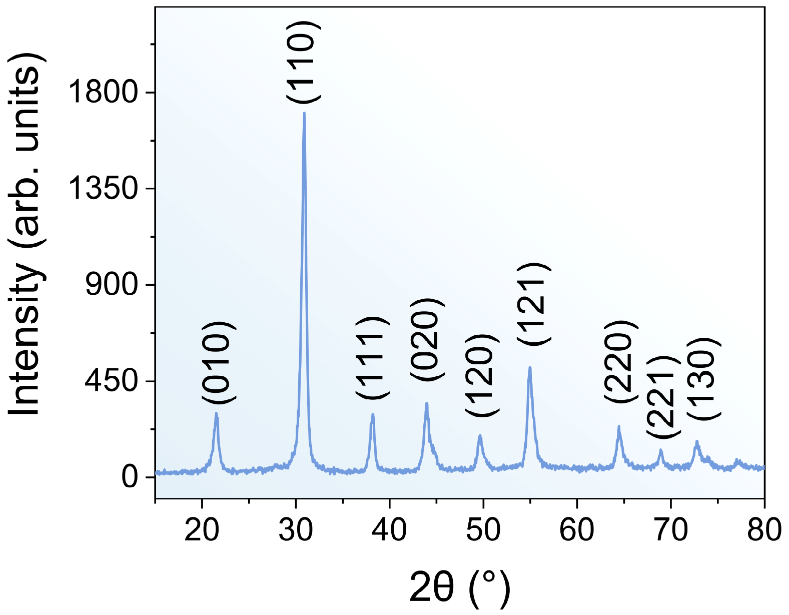

Supplementary Figure 1. X-ray diffraction pattern of PZT sample. The diffraction peaks at 21.6°, 30.9°, 38.2°, 44.0°, 49.7°, 55.3°, 64.5°, 69.0°, 72.8° correspond to the (010), (110), (111), (020), (120), (121), (220), (221), (130) crystal planes of PZT respectively. All reflection peaks agree well with the standard pattern for PZT (PDF no. 70-4060), indicating that the PZT material we use in the plasma reactor is of high purity.

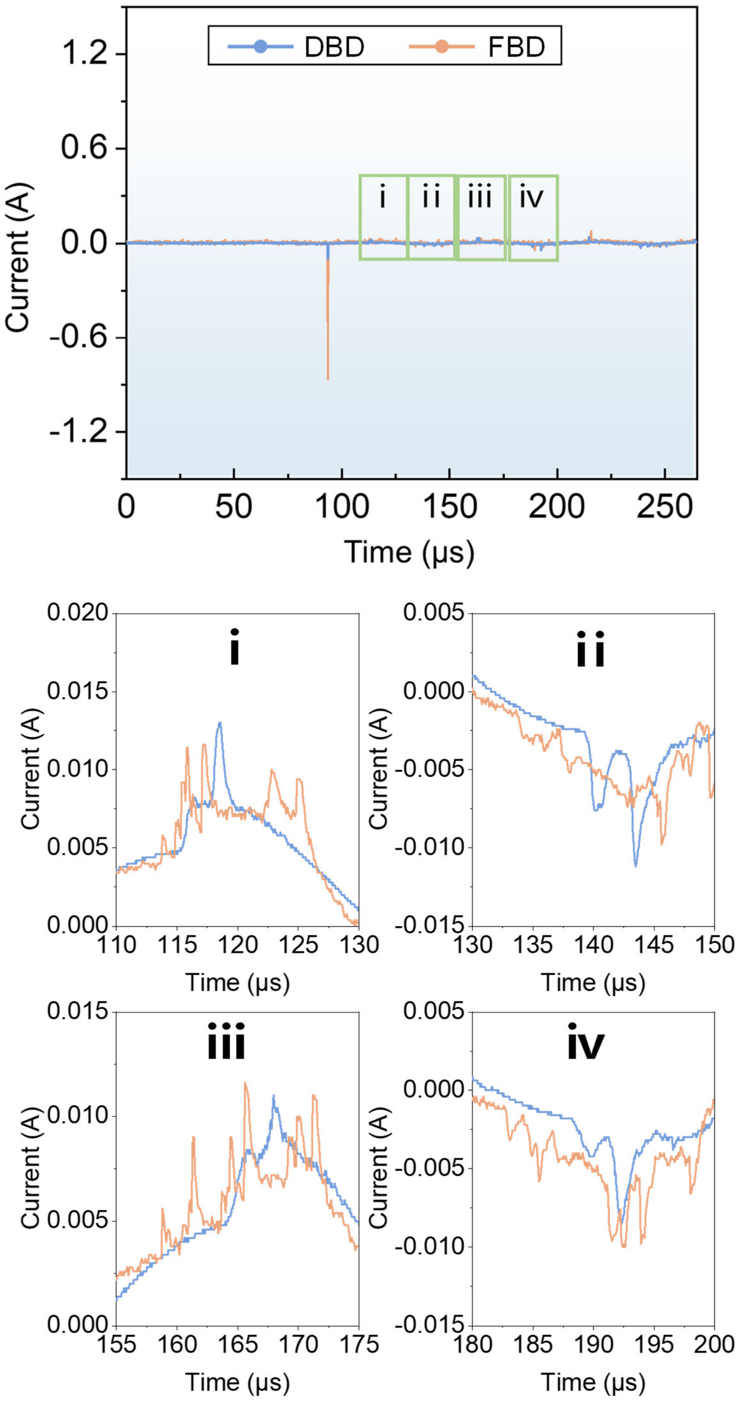

Supplementary Figure 2. The current waveforms of ferroelectric barrier discharge (FBD) and dielectric barrier discharge (DBD), with four zoom-in detailed subplots.

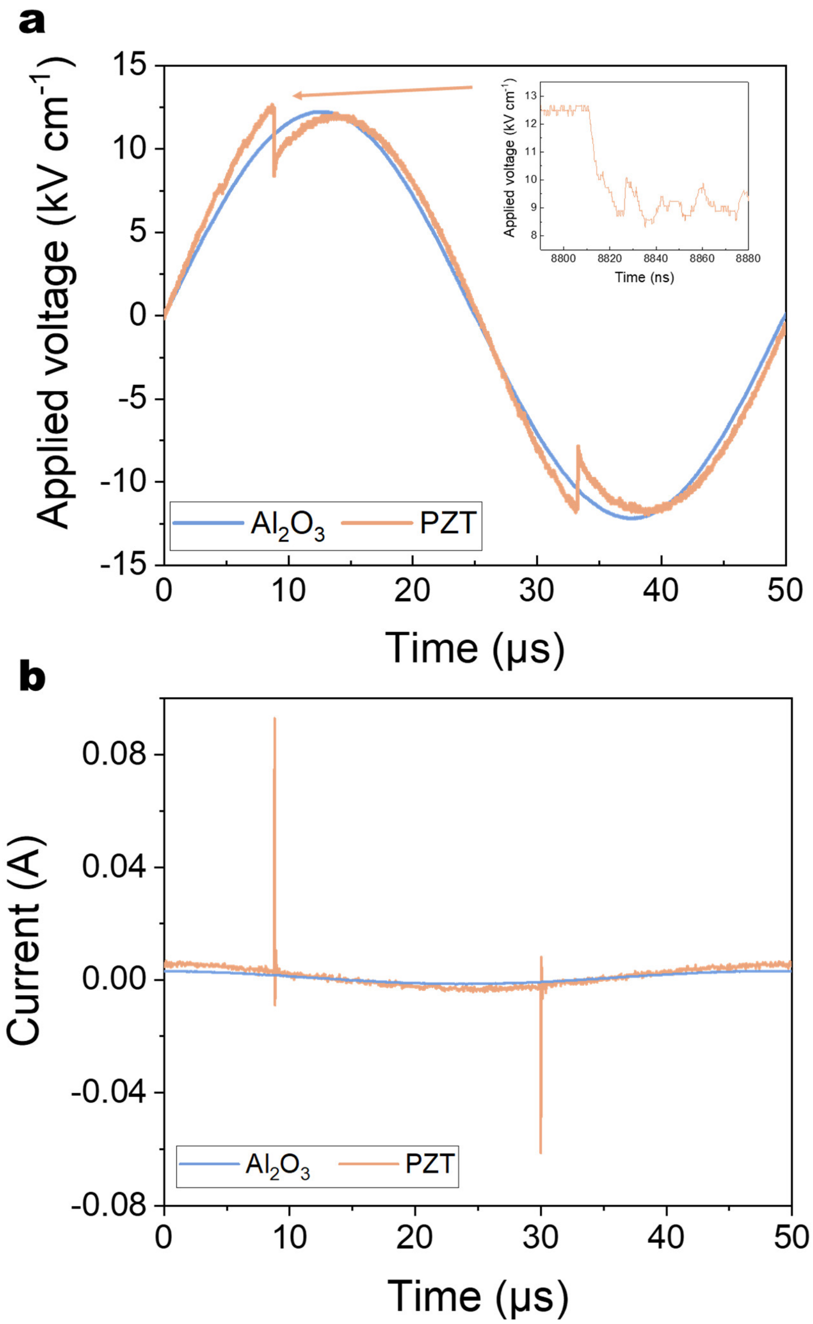

Supplementary Figure 3. Time-resolved ferroelectric switching measurements of $Pb(Zr_xTi_{1-x})O_3$ (PZT) and alumina samples: (a) applied voltage and (b) current. The timescale of ferroelectric polarization can be characterized by the fall time of the transient voltage change process, which is below 10 ns.

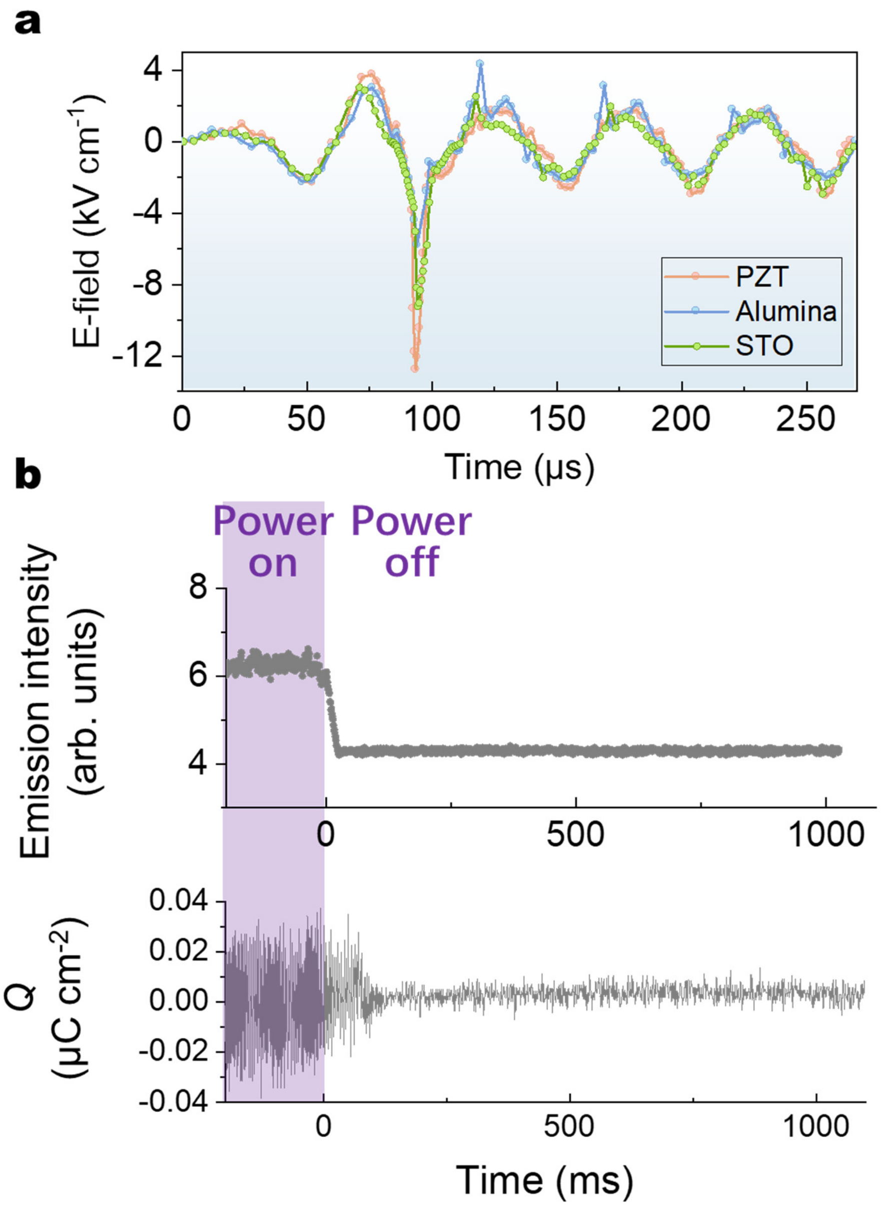

Supplementary Figure 4. The electric field and afterglow enhancements of the non-equilibrium plasma with $SrTiO_3$ (STO) as electrode barrier material. (a) The electric field measurements on ferroelectric barrier discharge based on PZT (with a dielectric constant of 1400), dielectric barrier discharge based on alumina (with a dielectric constant of 9), and STO (with a dielectric constant of 300) barrier discharge. (b) The time histories of STO barrier discharge plasma afterglow emission intensity and surface charge.

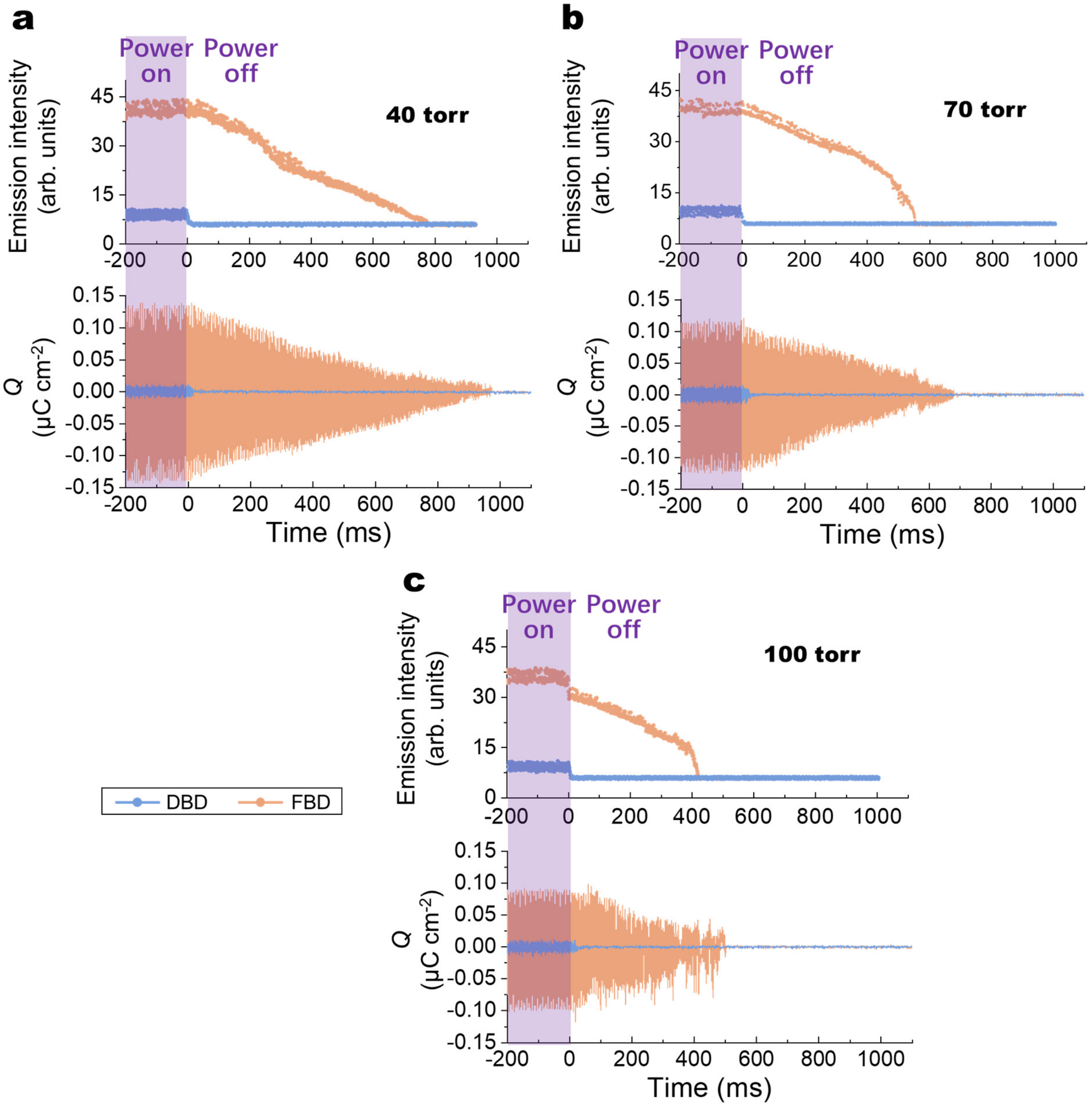

Supplementary Figure 5. The time histories of ferroelectric barrier discharge (FBD) and dielectric barrier discharge (DBD) afterglow emission intensity and surface charge at different pressures including (a) 40 torr, (b) 70 torr, and (c) 100 torr.

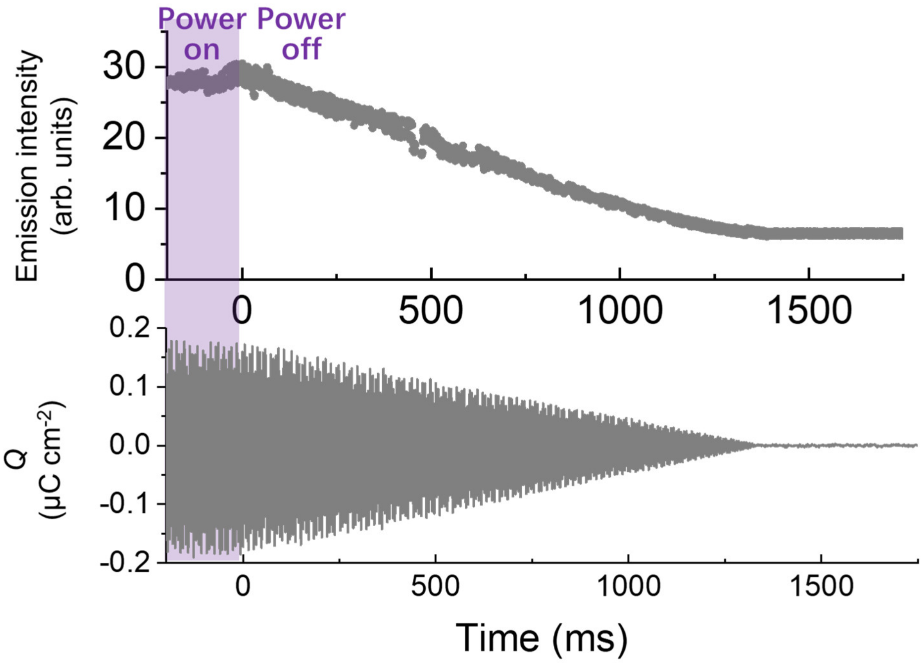

Supplementary Figure 6. The time histories of $BaTiO_3$ barrier discharge afterglow emission intensity and surface charge.

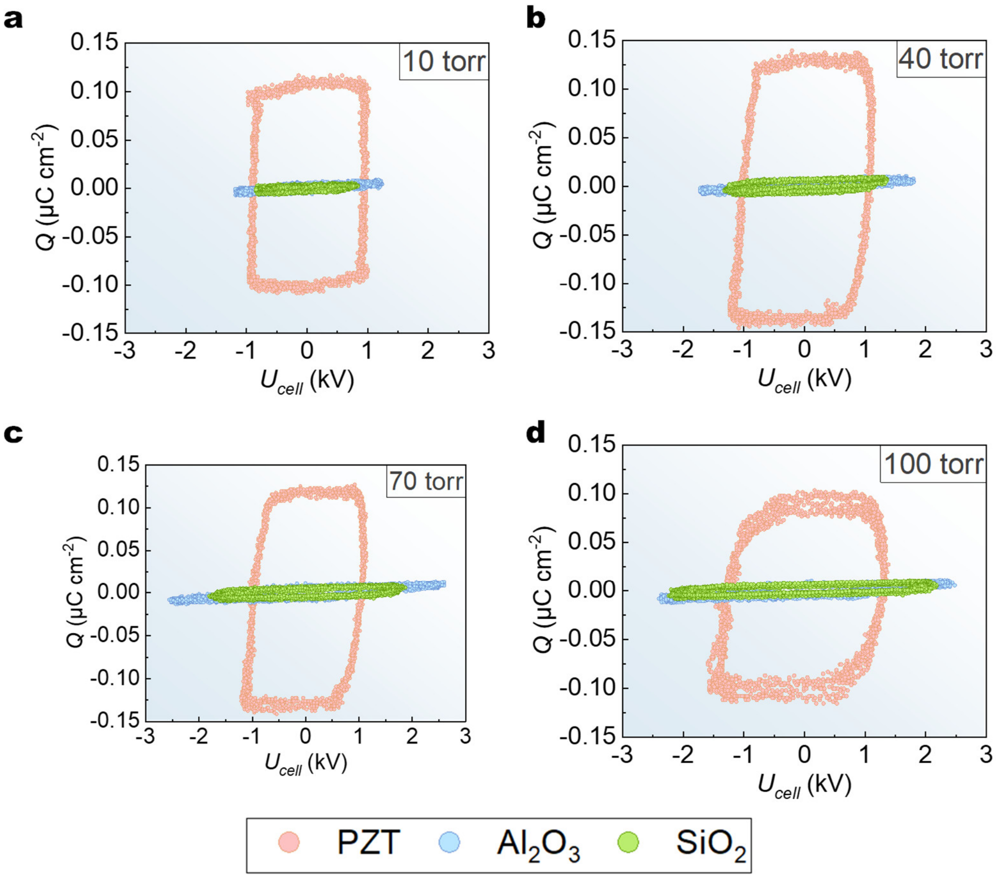

Supplementary Figure 7. Surface charge measurements of FBD, DBD and SiO_2 barrier discharge at (a) 10 torr, (b) 40 torr, (c) 70 torr, and (d) 100 torr.
