# Supplementary material for: Enhancements of electric field and afterglow of non-equilibrium plasma by Pb(ZrxTi1−x)O3 ferroelectric electrode
Source: Nat Commun. 2024 Apr 10;15:3092. doi: 10.1038/s41467-024-47230-7 (PMC11006859; doi:10.1038/s41467-024-47230-7)
Supplement: Supplementary file 3 — Reporting Summary [file 41467_2024_47230_MOESM3_ESM.pdf]

nature research

Corresponding author(s): Ning Liu

Lasing Reporting Summary

Nature Research wishes to improve the reproducibility of the work that we publish. This form is intended for publication with all accepted papers reporting claims of lasing and provides structure for consistency and transparency in reporting. Some list items might not apply to an individual manuscript, but all fields must be completed for clarity.

For further information on Nature Research policies, including our data availability policy, see Authors & Referees.

• Experimental design

Please check: are the following details reported in the manuscript?

1. Threshold

Plots of device output power versus pump power over a wide range of values indicating a clear threshold

- [ ] Yes
- [x] No

We did not change the output power of the lasers. Each laser experiment uses fixed output power as detailed in the manuscript.

2. Linewidth narrowing

Plots of spectral power density for the emission at pump powers below, around, and above the lasing threshold, indicating a clear linewidth narrowing at threshold

- [ ] Yes
- [x] No

All the laser experiments are not sensitive to linewidth narrowing.

Resolution of the spectrometer used to make spectral measurements

- [ ] Yes
- [x] No

We did not use any spectrometer.

3. Coherent emission

Measurements of the coherence and/or polarization of the emission

- [ ] Yes
- [x] No

This coherent emission does not affect the results in our work.

4. Beam spatial profile

Image and/or measurement of the spatial shape and profile of the emission, showing a well-defined beam above threshold

- [ ] Yes
- [x] No

All the laser experiments are not sensitive to beam spatial profile.

5. Operating conditions

Description of the laser and pumping conditions
Continuous-wave, pulsed, temperature of operation

- [x] Yes
- [ ] No

The detailed operation conditions have been described in the Methods section.

Threshold values provided as density values (e.g. W cm^{-2} or J cm^{-2}) taking into account the area of the device

- [ ] Yes
- [x] No

Again, we did not change the laser power, and so the threshold value is not critical.

6. Alternative explanations

Reasoning as to why alternative explanations have been ruled out as responsible for the emission characteristics
e.g. amplified spontaneous, directional scattering; modification of fluorescence spectrum by the cavity

- [x] Yes
- [ ] No

As mentioned in the Methods sections, we applied different filters (e.g., bandpass, longpass, etc.) to block any interferences in each laser experiment.

7. Theoretical analysis

Theoretical analysis that ensures that the experimental values measured are realistic and reasonable
e.g. laser threshold, linewidth, cavity gain-loss, efficiency

- [ ] Yes
- [x] No

Our laser experiments used very common operation conditions, and therefore the theoretical analysis to ensure the experiments are realistic and reasonable is not necessary.

8. Statistics

Number of devices fabricated and tested

- [x] Yes
- [ ] No

One laser.

Statistical analysis of the device performance and lifetime (time to failure)

- [ ] Yes
- [x] No

Our lasers used in the experiments are very stable, while experiments were done in seconds, not sensitive to time. Also, we monitored the laser pulse energy using a power meter and the results showed great stability of laser operations.

March 2018
